# Raf Activation Is Regulated by Tyrosine 510 Phosphorylation in *Drosophila*

**DOI:** 10.1371/journal.pbio.0060128

**Published:** 2008-05-20

**Authors:** Fan Xia, Jinghong Li, Gavin W Hickey, Amy Tsurumi, Kimberly Larson, Dongdong Guo, Shian-Jang Yan, Louis Silver-Morse, Willis X Li

**Affiliations:** Department of Biomedical Genetics, University of Rochester Medical Center, Rochester, New York, United States of America; Johns Hopkins University, United States of America

## Abstract

The proto-oncoprotein Raf is pivotal for mitogen-activated protein kinase (MAPK) signaling, and its aberrant activation has been implicated in multiple human cancers. However, the precise molecular mechanism of Raf activation, especially for B-Raf, remains unresolved. By genetic and biochemical studies, we demonstrate that phosphorylation of tyrosine 510 is essential for activation of *Drosophila* Raf (Draf), which is an ortholog of mammalian B-Raf. Y510 of Draf is phosphorylated by the c-src homolog Src64B. Acidic substitution of Y510 promotes and phenylalanine substitution impairs Draf activation without affecting its enzymatic activity, suggesting that Y510 plays a purely regulatory role. We further show that Y510 regulates Draf activation by affecting the autoinhibitory interaction between the N- and C-terminal fragments of the protein. Finally, we show that Src64B is required for Draf activation in several developmental processes. Together, these results suggest a novel mechanism of Raf activation via Src-mediated tyrosine phosphorylation. Since Y510 is a conserved residue in the kinase domain of all Raf proteins, this mechanism is likely evolutionarily conserved.

## Author Summary

Receptor tyrosine kinase (RTK)/Ras signaling pathways control many different biological processes during metazoan development. Mutations that disrupt this signaling pathway cause many human diseases, including cancer. The proto-oncoprotein Raf functions downstream of Ras in transducing signals from RTK. Activating mutations in both Ras and Raf have been linked to many types of human cancers. Despite the importance of these oncoproteins in tumorigenesis, the molecular mechanisms of Raf activation remains unresolved. Here, using a genetic screen in *Drosophila*, we show that the Src homolog Src64B is an activator of *Drosophila* Raf (Draf) .Src64B phosphorylates tyrosine Y510, in the Draf kinase domain and will activate a full-length Draf, but not a truncated Draf that contains only its kinase domain, suggesting that Y510 phosphorylation may relieve the autoinhibition of full-length Draf. Since Y510 is conserved among all the members of the Raf protein family, its phosphorylation may serve as a mechanism of Raf regulation in general.

## Abstract 3

Phosphorylation of a conserved tyrosine residue located in the kinase domain of Raf family proteins can serve as a mechanism of Raf activation.

## Introduction

The Raf serine/threonine kinase is a key component of the evolutionarily conserved signal transduction module that also includes the Ras GTPase, the mitogen and extracellular signaling-regulated kinase kinase (MEK), and the extracellular signaling-regulated kinase (ERK) [1,2]. In the canonical model, receptor tyrosine kinase (RTK) activation by extracellular signals such as peptide ligands leads to, via a series of adaptor proteins, the activation of Ras, which switches from GDP- to GTP-bound form. Ras-GTP binds to and thus causes the translocation of Raf to the plasma membrane, where it is activated by mechanisms that are still not completely resolved. It has been reported that somatic mutations in B-Raf are found in 60% of malignant melanomas and are also associated with other types of human cancers [3,4], which underscores the importance of this signaling pathway in tumorigenesis.

The mammalian Raf family consists of A-Raf, B-Raf, and C-Raf (also known as Raf-1 or c-Raf), which share three highly conserved regions (CR1–3; see Figure S1) [5,6]. The sole Raf homolog present in the *Drosophila* genome, Draf, is encoded by *lethal (1) pole hole* (*phl*); it contains all three conserved domains and is most homologous to B-Raf and secondarily to C-Raf, with 61% and 54% overall sequence similarity, respectively [7,8]. Draf shares similar substrate specificity and regulatory mechanisms with C-Raf and very likely also with B-Raf, as expression of human C-Raf in Draf mutants can restore Draf signaling [9,10]. CR1 and 2 are located in the N-terminal regulatory region, which acts to repress the catalytic activity of kinase domain located in the C-terminal CR3. Two subdomains in CR1, a Ras-binding domain (RBD) and a cysteine-rich domain (CRD), are responsible for binding to Ras-GTP [5,6].

The mechanism of Raf activation is complex and, to date, not fully understood. Numerous factors, including Ras, Ksr, CNK, 14-3-3, Src, and others, have been reported to regulate Raf activation [5,6,11]. Binding to Ras-GTP, an important first step leading to Raf activation, serves to translocate Raf to the membrane and subject Raf to activation by other factors localized at the membrane [12]. Following Ras binding, modifications of Raf by phosphorylation that occur at the plasma membrane appear essential for Raf activation [13,14] (Figure S1). C-Raf is mainly phosphorylated on S338, Y341, T491, and S494 following Ras-induced activation, and numerous factors have been implicated in phosphorylating these sites [6,11]. S338 and Y341 reside in the “N region,” a negatively charged regulatory region located at the N-terminus of the kinase domain, about 20 amino acids from the ATP-binding domain [15,16]. Phosphorylation of these sites is believed to play a role in relieving the inhibitory N-terminus from the C-terminal kinase domain [17,18]. The p21-activated kinase 3 (PAK3) has been identified as a kinase capable of phosphorylating S338 [19,20]. Y341 is phosphorylated as a result of overexpression of activated Src family kinase (SFK) Src in cultured cells and in vitro, and substitution of this residue with the phosphomimetic aspartate increases C-Raf activity [15,21–23]. In addition, it has also been shown that Src activity is required for rapid activation of mitogen-activated protein kinase (MAPK) signaling [24], and that Src can function downstream of RTK to induce Shc/Grb association, leading to Ras activation [25,26]. However, studies of mouse cells lacking three SFKs (Src, Yes, and Fyn) indicate that SFKs are mostly dispensable for RTK signaling [27]. Thus, the interplay between SFKs and RTKs is complex, and whether endogenous Src plays a direct role in Raf activation remains unclear.

In addition to the N region, T491 and S494 in the activation segment of the kinase domain are also phosphorylated in a Ras-dependent manner [16]. Recent studies of B-Raf suggest that phosphorylation on these activation loop residues may be important for catalytic activity and/or stabilization of the active conformation of the kinase [28]. However, the kinases responsible for their phosphorylation remain elusive. Interestingly, in B-Raf, the equivalent of S338 is constitutively phosphorylated and the equivalent of Y340/341 are occupied by aspartic acids; consequently, B-Raf exhibits higher basal activity and was shown not to be regulated by Src [15]. Similar to B-Raf, Draf also contains two acidic residues (glutamates) at positions equivalent to Y340/341 of C-Raf, and the equivalent to S338 in C-Raf is also constitutively phosphorylated [29] (Figure S1). Thus, it has been unclear how B-Raf or Draf are regulated in vivo.

In an effort to identify potential activators of Draf, we have previously conducted a genetic screen for genes involved in Draf signaling, taking advantage of a *Draf* hypomorphic allele, *Draf^Su2^*, which causes partial lethality to flies [30]. *Draf^Su2^* encodes a Draf variant with two point mutations that abolish its Ras-binding ability, making it more sensitive to reductions in Ras-independent Draf activators [8,30]. This genetic screen identified Src64B as a potential Draf activator, as reducing the gene dosage of *Src64B* dominantly enhanced the lethality associated with *Draf^Su2^* flies [30]. To determine the role of Src64B in Draf activation in vivo, we genetically and biochemically investigated the function of Src64B in Draf activation. Here, we show that Src64B behaves as a direct Draf activator in vivo. An activated form of Src64B induces Draf target genes in the absence of RTK or Ras in vivo, and associates with and phosphorylates Draf. Moreover, we identified a novel tyrosine (Y510) within the Draf kinase domain that mediates Draf phosphorylation by Src64B in vitro. Interestingly, the role of Y510 appears to be purely regulatory, as mutating it to phenylalanine or glutamate had no significant impact on the enzymatic activity of a Draf kinase domain fragment. However, mutating Y510 to glutamate resulted in activation of full-length Draf and reduced affinity between N- and C-terminal Draf fragments. These results suggest that Y510 phosphorylation plays an essential role in Draf activation by interfering with the association of the C-terminal kinase domain with the inhibitory N-terminal regulatory region.

## Results

### Src64B Can Function Downstream or in Parallel to Ras1

**Figure 1:**
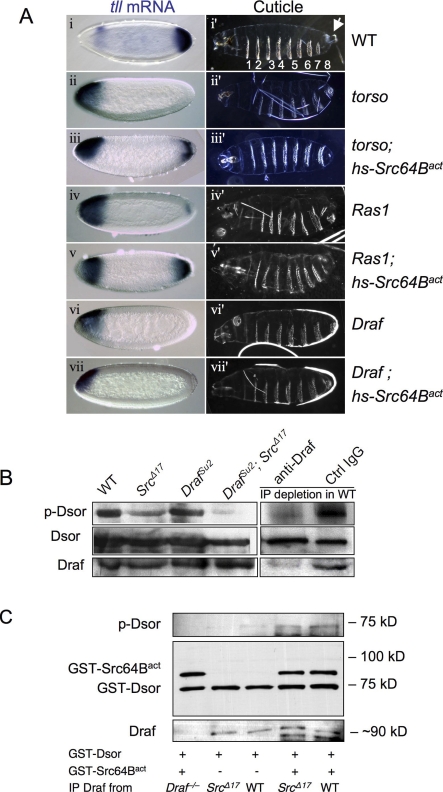
Src64B Can Function Downstream or in Parallel to Ras in Activating Draf (A) Expression of *tll* mRNA (blue stains) in stage 4 embryos (left column) was detected by in situ hybridization. The right panels show cuticles of mature embryos. All the embryos are shown with anterior to the left. Genotypes are noted to the right. *Draf* indicates *Draf^11−29^; Ras1*, *Ras1^ΔC40B^;*and *torso*, *torso^XR1^*. (i) *tll* is expressed in anterior and posterior regions of wild-type (WT) embryos. The posterior *tll* expression is solely dependent on the Torso pathway; the anterior *tll* expression is repressed by Torso signaling and serves as an internal control. (i′) Wild-type cuticles exhibit eight ventral denticle belts (numbered 1–8) as well as head and tail (terminal) structures. The posterior terminal structures include the eighth ventral denticle belt and the Filzkörper (arrow), which require appropriate posterior *tll* expression. Embryos maternally null for *torso* (ii and ii′), *Ras1* (iv and iv′), or *Draf* (vi and vi′) are missing posterior *tll* expression, and as a consequence, are missing posterior structures and exhibit only seven ventral denticle belts. Transient expression of Src64B^act^ during early embryogenesis restored the posterior *tll* expression and the eighth denticle band in *torso* (iii and iii′) or *Ras1* (v and v′) mutant embryos, but not in *Draf* mutants (vii and vii′). More than 200 embryos were observed for each genotype. Representative pictures are shown. (B) The activity of the Ras-binding–deficient Draf^Su2^ variant is dependent on Src64B. Cell-free extracts from embryos of indicated genotypes were subjected to in vitro kinase assay using purified GST-Dsor1 (MEK homolog) as substrate. Note Draf^Su2^ activity is dramatically decreased in *Src64B^Δ17^* background (cf. lanes 3 and 4). Wild-type Draf activity is only mildly decreased in *Src64B^Δ17^* background (cf. lanes 1 and 2). Depletion of Draf by specific antibody abolished the Dsor1 phosphorylation (lane 5). (C) Draf was immunoprecipitated from embryos of indicated genotypes. *Draf^−/−^* embryos were maternally null for *Draf* (negative control). The immunoprecipitates were subjected to kinase assay using GST-Dsor as substrate with or without added Src64B^act^. Note the kinase activity of Draf from *Src64B^Δ17^* was undetectable (lane 2). Addition of Src64B^act^ increased the activity of Draf from both *Src64B^Δ17^* and wild-type embryos to similar levels (lanes 4 and 5).

To investigate whether Src64B plays a direct role in Draf activation, we first tested whether it can induce Draf activation in the absence of Ras1. We examined the effects of expressing an activated form of Src64B (referred to as Src64B^act^; a.k.a. Src64B^Δ540^) [31] on activities of the Torso-Ras1-Draf signaling pathway using the Torso target gene *tailless* (*tll*) as a readout [32,33]. Src64B^act^ (or Src64B^Δ540^) is truncated from amino acid (a.a.) 540 and lacks the C-terminal autoinhibitory domain [31]. The posterior expression domain of *tll* in the early embryo reflects quantitatively the strength of Torso or Draf activation [8,33–35]. *tll* is expressed from 0% to 15% of egg length (EL) from the posterior in wild-type embryos and is absent or little detected in embryos lacking Torso, Draf, or Ras1 (Figure 1A; also see Hou et al., 1995 [35]; Li et al., 1997 [34]; see Materials and Methods for mutant embryo production). We have previously shown that heat-shock induction of Src64B^act^ can induce ectopic *tll* expression in wild-type embryos [30]. If Src64B plays a role in activating Draf, we expect that Src64B^act^ would induce *tll* expression in the absence of Torso or Ras1, but not Draf. Indeed, we found that a brief induction of Src64B^act^ expression in the early embryos was capable of inducing expression of *tll* in embryos lacking Torso or Ras1, but not in those lacking Draf (Figure 1A). Consistent with its effects on *tll* expression, expression of *Src64B^act^* was able to rescue to a certain extent the cuticular defects associated with *torso* or *Ras1*, but not *Draf* mutant embryos (Figure 1A). It has been shown that activated Src can induce the formation of a complex between Shc and Grb2, an event upstream of Ras in the activation of this signaling pathway [25], and that the effects of Src64B^act^ on eye differentiation are sensitive to the dosage of Ras1 [31]. However, our results indicate that Src64B is able to function downstream or in parallel to Ras1 but upstream of Draf, suggesting it might be able to directly activate Draf.

### Src64B Is Required for Draf Kinase Activity

To test whether endogenous Src64B is required for Draf activation in vivo, we sought to analyze the phenotypes of existing mutant alleles of *Src64B*. *Src64B^Δ17^* and *Src64B^PI^* are the strongest mutant alleles isolated to date, although both are hypomorphs [36]. Homozygous mutant animals for these alleles exhibit defects in oogenesis [36]. We focused on *Src64B^Δ17^*, which is associated with a deletion that removes the first two noncoding exons of *Src64B* [36]. Other than the oogenesis defects, which result in partial female sterility, *Src64B^Δ17^* homozygotes are morphologically normal and exhibit no discernable developmental or behavioral defects (unpublished data; also see [36]), and all aspects of RTK signaling examined appear normal (see below). This suggests that the residual activity in the mutant suffices for development, or that Src64B is functionally redundant.

The role of Ras in Raf activation is mainly attributed to Ras binding to the inhibitory N-terminus of Raf and targeting Raf to the membrane [8,37,38]. Since the Draf allele, *Draf^Su2^*, harbors point mutations that abolish its interaction with Ras [8], it is thus an ideal tool to test whether Src64B is required to directly activate Draf in vivo. To determine whether the activation of Draf, especially the activation of Draf^Su2^, is impaired in *Src64B* mutants, we measured Draf kinase activity in protein extracts from different mutant combinations. Using protein extracts and in vitro kinase assays with the downstream suppressor of Raf 1 (Dsor1; a MEK homolog) as substrate, we found that extracts from *Src64B^Δ17^* homozygotes exhibited reduced levels of Draf activity (Figure 1B, lane 2), and the reduction in Draf kinase activity was even more pronounced in extracts from *Draf^Su2^*; *Src64B^Δ17^* double homozygotes (Figure 1B, lane 4). The Draf activity detected in fly embryo extracts in vitro was indeed dependent on the presence of Draf, as depletion of Draf from the protein extracts prior to kinase assay by an anti-Draf antibody abolished Dsor1 phosphorylation (Figure 1B, lane 5). Thus, even though *Src64B* hypomorphic mutants are morphologically normal, their Draf activity is severely compromised.

We next determined whether addition of Src64B^act^ could restore the activity of Draf immunoprecipitated from *Src64B^Δ17^* mutant embryos. Draf immunoprecipitated from *Src64B^Δ17^* and wild-type fly embryos had undetectable and low kinase activities, respectively (Figure 1C, lanes 2, and 3). However, addition of bacterially purified Src64B^act^ (see Figure S2 for activity) significantly increased the kinase activities of immunoprecipitated Draf proteins from *Src64B^Δ17^* and wild-type embryos to comparable levels (Figure 1C, lanes 4 and 5). Src64B^act^ did not directly cause Dsor1 phosphorylation (Figure 1C, lane 1). A kinase-dead version of Src64B^act^ had no effect on immunoprecipitated Draf (unpublished data). These results suggest that the deficit in Draf kinase activity exhibited by *Src64B^Δ17^* flies might be due to reduced phosphorylation by Src64B.

Finally, consistent with the idea that Src64B activation increases Draf activity in vivo, we found that expressing Src64B^act^ rescued the lethality associated with *Draf^C110^* hemizygous males (Figure S3). *Draf^C110^* is a hypomorphic allele, and *Draf^C110^* hemizygous males die as late pupae [7]. In summary, the above independent pieces of evidence suggest that Src64B is required for Draf activation in vivo, that activated Src64B can mediate Draf activation independent of Ras1, and that Src64B might directly activate Draf by phosphorylation.

### Src64B Binds to and Phosphorylates Draf on Y510

**Figure 2:**
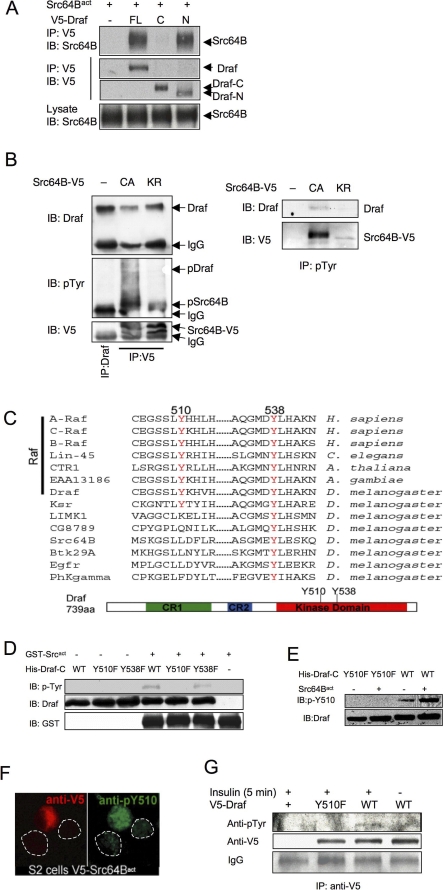
Src64B Binds to Draf and Phosphorylates Draf on Y510 (A) Src64B binds to Draf mainly through the N-terminal region. Src64B^act^ was cotransfected with full-length Draf (FL), Draf-N (N), or Draf-C (C) into S2 cells. Transfected Draf was immunoprecipitated (IP) with anti-V5 and blotted (IB) with anti-Src64B. Note Src64B^act^ was coimmunoprecipitated with Draf or Draf-N, but very little with Draf-C. (B) Src64B binding leads to tyrosine phosphorylation of Draf. S2 cells were transfected with V5-tagged Src64B^act^ (referred to as CA, constitutive active) or a kinase-dead version (Src64B^KR^; KR), immunoprecipitated with anti-V5 (left) or anti-pTyr (right), and subjected to SDS-PAGE. Immunoprecipitated Draf from untransfected S2 cells was used as input to mark the position of Draf (left panels, lane 1). Note that the endogenous Draf was co-immunoprecipitated with transfected Src proteins (left panels; lanes 2 and 3). Transfection of Src64B^act^ (CA), but not Src64B^KR^, led to tyrosine phosphorylation of coimmunoprecipitated Draf (middle left). Also note that the endogenous Draf was immunoprecipitated by anti-pTyr only when S2 cells were transfected with Src64B^act^, but not Src64B^KR^ (right panels). (C) Conservation of Y510 and Y538 in protein kinases. A multisequence alignment of several kinases identifies two conserved tyrosine residues in the kinase domain, which correspond to Y510 and Y538 of Draf (red). Y510 is conserved in all Raf family members as well as in Ksr, whereas Y538 is conserved in all kinases analyzed, including tyrosine kinases such as EGFR and Src64B. The positions of Y510 and Y538 are also indicated in the schematic representation of Draf. (D–F) Src64B phosphorylates Draf-C on Y510. (D) Src64B phosphorylates Draf-C on Y510 in vitro. GST-Src64B^act^, His-Draf-C^WT^, His-Draf-C^Y510F^, and His-Draf-C^Y538F^ proteins purified from E. coli were subjected to in vitro kinase assay. The levels of tyrosine phosphorylation on different Draf-C molecules were detected by an anti-pTyr antibody. Increased tyrosine phosphorylation levels in the presence of GST-Src64B^act^ were detected for His-Draf-C^WT^ (lane 4) and His-Draf-C^Y538F^ (lane 6), but not for His-Draf-C^Y510F^ (lane 5). WT, wild type. (E) In vitro kinase assay was carried out as in (D), and the proteins were subjected to SDS-PAGE and blotted with an antibody specific for phospho-Y510 of Draf (pY510). Note that anti-pY510 recognized Draf-C^WT^ (lane 4), but not Draf-C^Y510F^ (lane 2), following incubation with GST-Src64B^act^. (F) Transfection of V5-Src64B^act^ (red; anti-V5) into S2 cells resulted in increased Y510 phosphorylation of endogenous Draf (green; anti-pY510). Dotted lines circle two adjacent untransfected cells. (G) Y510 phosphorylation correlates with Draf activation in S2 cells. Five minutes following insulin addition, V5-tagged Draf proteins were immunoprecipitated and then subjected to western blots with anti-pTyr. Note that tyrosine phosphorylation was detected in Draf^WT^ (lane 3), but not Draf^Y510F^ (lane 2).

To investigate the mechanism of Src64B-mediated Draf activation, we first determined whether Src64B binds to or phosphorylates Draf. By transfection and coimmunoprecipitation experiments, we found that Src64B^act^ is indeed able to bind to Draf, mainly to the N-terminal half, and very weakly to the C-terminus (Figure 2A). Moreover, we found that when expressed in *Drosophila* S2 cells, both Src64B^act^ and its kinase-dead version Src64B^KR^ were able to bind to endogenous Draf, as the endogenous Draf was coimmunoprecipitated with transfected Src64B molecules (Figure 2B). However, tyrosine phosphorylation was detected only in Draf coimmunoprecipitated with Src64B^act^, suggesting that Src64B^act^ can directly phosphorylate Draf (Figure 2B). Indeed, using bacterially expressed proteins and in vitro kinase assays, we found that GST-Src64B^act^ phosphorylated a C-terminal Draf fragment, Draf-C, consisting of the kinase domain (see below). It has previously been reported that Fyn/Src binds to and phosphorylates C-Raf on tyrosine residues [39]. Interestingly, unlike other Src substrates that bind to Src through SH2-phosphotyrosine interaction, binding to C-Raf by the SH2 domain of Src does not require phosphotyrosine residues on C-Raf [39]. Our results are consistent with this finding and suggest that Src64B associates with Draf N-terminal region in a phosphotyrosine-independent manner and that Src64B can phosphorylate Draf.

We next searched for candidate tyrosine residues in Draf that can be phosphorylated by Src64B^act^. Sequence comparison revealed two tyrosine residues within the kinase domain (Y510 and Y538) that are conserved among all Raf proteins (Figure 2C). We mutated these tyrosine residues to phenylalanine, a non-phosphorylatable neutral substitution for tyrosine, and tested whether any of the mutations would affect Draf phosphorylation by Src64B. By comparing tyrosine phosphorylation levels of Draf-C^WT^ with those of Draf-C^Y510F^ and Draf-C^Y538F^ in the presence or absence of GST-Src64B^act^, we found that the Y510F substitution greatly diminished phosphorylation by GST-Src64B^act^, whereas Y538F had little effect (Figure 2D). Since Src64B binds to Draf mainly through the N-terminal region (Figure 2A) and Src can bind to Raf independent of phosphotyrosine residues (Figure 2B) [39], Y510 of Draf-C is unlikely responsible for binding with Src64B. Thus, a plausible explanation for the above results is that Y510 is the major Src64B phosphorylation site on Draf.

We confirmed that Src64B phosphorylates Draf on Y510 by producing polyclonal antibodies specific for phospho-Y510 of Draf (anti-pY510; see Materials and Methods and Figure S4). Indeed, the anti-pY510 antibody recognized phosphorylated Draf^WT^, but not Draf^Y510F^, following incubation with Src64B^act^ (Figure 2E). Consistent with the in vitro kinase assay data, pY510 levels were increased in Src64B^act^-transfected S2 cells (Figure 2F), suggesting Src64B can phosphorylate endogenous Draf on Y510 in vivo.

To investigate whether phosphorylation of Y510 in Draf correlates with Draf activation in vivo, we examined the phosphorylation status of Draf immediately following its activation in S2 cells. It has previously been shown that S2 cells respond to insulin stimulation by activating Draf [40]. Immediately following insulin stimulation, we detected tyrosine phosphorylation, albeit low, in transfected Draf^WT^, but not Draf^Y510F^ (Figure 2G). It has previously been shown for C-Raf that activated Raf, which resides in the membrane and is tyrosine phosphorylated, constitutes only a minority of total Raf proteins [13,14]. This may explain the difficulty to detect tyrosine phosphorylation of Raf proteins. Taken together, these results suggest that Y510 becomes phosphorylated upon Draf activation.

### Substituting Tyr510 with Glutamate Activates Full-Length Draf In Vitro

**Figure 3:**
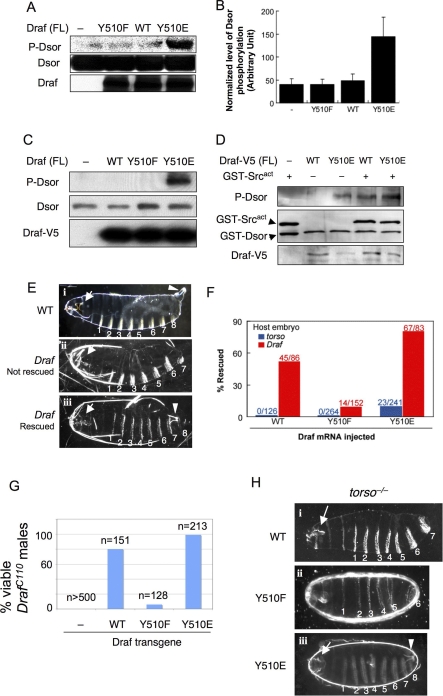
Acidic Substitution of Y510 Promotes and Phenylalanine Substitution Impairs Activation of Full-Length Draf In Vitro and In Vivo (A) Draf^Y510E^ (lane 4) exhibited dramatically higher activities by in vitro kinase assay using bacterially expressed full-length Draf variants and GST-Dsor1. Dsor1 phosphorylation was detected by anti-pMEK. WT, wild type. (B) Quantification of results represented in (A). (C) V5-tagged full-length Draf variants were transfected into S2 cells and were immunoprecipitated with anti-V5 and subjected to kinase assay using bacterially expressed Dsor1 as substrate. Note that only Draf^Y510E^ exhibited prominent kinase activity toward purified Dsor1, as detected by anti-pMEK (lane 4). (D) V5-tagged full-length Draf^WT^ or Draf^Y510E^ were transfected into S2 cells and were immunoprecipitated with anti-V5 and then subjected to kinase assay using bacterially expressed Dsor1 as substrate with or without purified GST-Src64B^act^. Note that GST-Src64B^act^ stimulated the activity of Draf^WT^ to that of Draf^Y510E^ (cf. lanes 3 and 4). (E–H) Effects of Y510 substitutions on Draf activity in vivo. (E and F) Injection of mRNA into early stage embryos. (i) Cuticles of a wild-type embryo exhibiting normal head skeleton (arrow), eight ventral denticle belts (numbered), and the Filzkörper (arrowhead). (ii) Cuticles from a buffer-injected (control) *Draf* ^−/−^ embryo exhibit characteristic *Draf* null phenotypes: collapsed head skeletons (arrow) and loss of all posterior structures (eighth denticle belt and the Filzkörper). (iii) A *Draf* null embryo rescued by injecting Draf^Y510E^ mRNA. Note the restored eighth denticle belt and the Filzkörper (arrowhead). Due to the limited diffusion of injected mRNA or different threshold requirement, the anterior (head skeleton; arrow) defects were not rescued, which serves as an internal control. The phenotypes of rescued *torso* null embryos are identical to *Draf* null embryos (unpublished data). (F) Draf^Y510E^ expressed from injected mRNA exhibited higher basal and inducible activity than Draf^WT^ (*p* < 0.001) in vivo. Draf^Y510F^ had impaired inducible activity (*p* < 0.001). Basal Draf activity was measured by the percentage of rescue of posterior structures of *torso* null embryos by mRNA microinjection (shown in blue). Draf^Y510E^ rescued 9.5% of injected *torso* embryos, whereas no rescue was found for Draf^Y510F^ and Draf^WT^. Inducible Draf activity was measured by the percentage of rescue of posterior structures of *Draf* null embryos by mRNA microinjection (shown in red). Draf^WT^, Draf^Y510F^, or Draf^Y510E^ rescued 52%, 9.2%, or 81% of Draf null embryos, respectively. The number of injected embryos scored is indicated. Chi-square tests were used to compare the differences, *p*-values are indicated. (G and H) In vivo activities of Draf transgenes with different Y510 substitutions. (G) Virgin *Draf^C110^/FM7* females were mated with males with a recombinant chromosome carrying *hsp70-Gal4* and an indicated *UAS-Draf* transgene. Surviving F1 *Draf^C110^*/Y males were scored and normalized as percent viability relative to control crosses. Note that *Draf^C110^*/Y males were not viable in the absence of *UAS-Draf* transgenes, but were rescued to different degrees by different Draf transgenes. (H) Females of *Nanos-Gal4; torso^XR1^; UAS-Draf* variants were mated to wild-type males, and the F1 progeny were examined for viability and cuticle patterns. Note that expressing Draf^WT^ or Draf^Y510F^ did not rescue *torso^XR1^* phenotypes, whereas expressing Draf^Y510E^ fully rescued *torso^XR1^* female sterility (unpublished data) or restored the posterior cuticle structures.

To explore whether Y510 is important for Draf activation, we measured the kinase activity of purified Draf proteins with different amino acid substitutions at Y510. An acidic substitution can mimic the effects of tyrosine phosphorylation by its negative charge [17,21,41–43]. We found that full-length Draf proteins with different Y510 substitutions exhibited different kinase activities when purified from bacteria. Whereas Draf^Y510F^ and Draf^WT^ exhibited no and barely detectable kinase activities, respectively, Draf^Y510E^, however, exhibited dramatically higher kinase activities (Figure 3A and 3B). Similar results were found when full-length Draf^WT^, Draf^Y510F^, and Draf^Y510E^ proteins were expressed in and immunoprecipitated from S2 cells (Figure 3C). Thus, acidic substitution of Y510 results in activation of full-length Draf.

To confirm that Y510E mimics Src64B phosphorylation, we subjected Draf^WT^ and Draf^Y510E^ immunoprecipitated from S2 cells to added Src64B^act^. We found that Src64B^act^ greatly stimulated the kinase activity of Draf^WT^ but hardly affected that of Draf^Y510E^ (Figure 3D).

### Draf^Y510E^ Behaves as an Activated and Draf^Y510F^ as a Dominant-Negative Kinase In Vivo

To investigate the activities of Draf with different Y510 substitutions in vivo in a developmental context, we expressed these molecules in early *Drosophila* embryos by mRNA injection. mRNA injection into early embryos has been used as a robust assay for functions of expressed proteins, including Draf [8,44]. Based on the ability of injected Draf mRNA to rescue the mutant phenotypes of *Draf* or *torso* null embryos (see Materials and Methods for mutant embryo production), the basal and inducible activities of different Draf proteins expressed can be quantitatively assessed [8,44]. Wild-type *Drosophila* embryos develop distinct cuticle patterns with eight abdominal ventral denticle bands and a prominent “tail” (Filzkörper; Figure 3E i, arrowhead). The posterior structures (A8 and Filzkörper) are specified by the Torso RTK signal transduction pathway, mediated by the Ras1/Draf signaling cassette [33], and *torso* or *Draf* null embryos fail to develop these posterior structures (Figure 3E ii).

We injected mRNA encoding each version of full-length Draf into *torso* embryos, in which the endogenous Draf remains inactive, and found that Draf^Y510E^ restored the posterior structures in 9.5% of injected *torso* embryos, whereas no rescue was found for Draf^Y510F^ and Draf^WT^ (Figure 3E iii and 3F). This suggests that Draf^Y510E^ has higher basal activity (*p* < 0.001), which is consistent with the in vitro kinase assay data. When injected into *Draf* embryos, which are devoid of the endogenous Draf, Draf^Y510E^ exhibited the highest rescuing activity, followed by Draf^WT^ and Draf^Y510F^ (Figure 3F). These results demonstrate that acidic substitution of Y510 results in Draf activation in vivo and a conserved substitution to a non-phosphorylatable residue renders Draf recalcitrant to activation.

To further confirm the mRNA injection results above, we generated transgenic flies carrying the three versions of full-length Draf transgenes under the control of the *Gal4*-inducible promoter (UAS; [45]). First, we tested the ability of each Draf transgene to rescue the lethality of flies hemizygous for the hypomorphic allele *Draf^C110^* [7]. We found that, when expressed at low levels by the basal activity of *hsp70-Gal4*, Draf^Y510E^ and Draf^WT^ rescued 99% and 80%, respectively, of the *Draf^C110^* hemizygotes, whereas Draf^Y510F^ had very low ability to do so (Figure 3G). Upon heat-shock induction, Draf^Y510E^, but not Draf^WT^, caused lethality (unpublished data). Second, we used a maternal *Gal4* driver (see Materials and Methods) to express the Draf transgenes in a *torso* null background and examined the cuticle structures of the resulting embryos. We found that expressing Draf^WT^ had no effect on *torso^−/−^* embryos (Figure 3H i), and expressing Draf^Y510F^ worsened the *torso^−/−^* phenotypes (Figure 3H ii), suggesting that Draf^Y510F^ may have dominant-negative effects. In contrast, expressing Draf^Y510E^ had dramatic effects, resulting in rescuing *torso^−/−^* embryos even to full viability, such that 68% of these embryos (*n* = 86/127) hatched to crawling larvae that subsequently developed to morphologically normal adults (unpublished data). Although the rest of the embryos failed to hatch, they exhibited wild-type posterior structures, with normal-appearing A8 and the Filzkörper (Figure 3H iii, arrowhead). Finally, we expressed these Draf transgenes in the developing eye and found that indeed Draf^Y510E^ behaved like an activated and Draf^Y510F^ a dominant-negative form of Draf (see below). These results strongly suggest that Draf^Y510E^ possesses elevated levels of kinase activity that are sufficient to overcome the lack of an upstream receptor Torso.

### Y510 Phosphorylation Interferes with Draf Autoinhibitory Interaction

**Figure 4:**
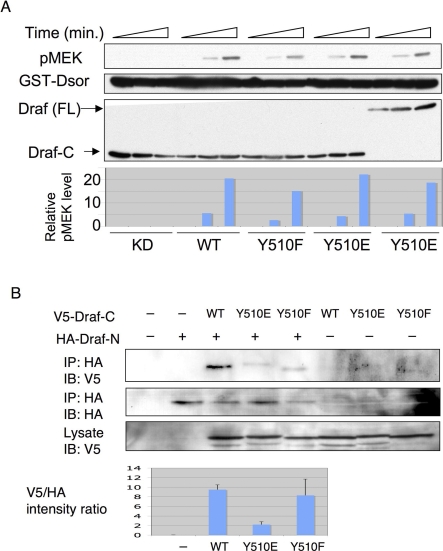
Y510E Substitution Interferes with Draf Autoinhibitory Interaction (A) V5-tagged Draf-C^KD^ (K497M; kinase-dead), Draf-C^WT^, Draf-C^Y510F^, Draf-C^Y510E^, and the full-length Draf^Y510E^ (FL) were transfected into S2 cells and immunoprecipitated by anti-V5. The immunoprecipitates were subjected to kinase assay using bacterially expressed Dsor1 as substrate for 0, 5, or 30 min, respectively. The kinase activity was measured by anti-pMEK signals and plotted at the bottom. Note that all Draf-C variants and the full-length Draf^Y510E^ exhibited similar kinetics and activities. WT, wild type. (B) V5-tagged Draf-C^WT^, Draf-C^Y510F^, or Draf-C^Y510E^ was cotransfected with or without HA-Draf-N (HA) into S2 cells, and the cells were subjected to immunoprecipitation (IP) by anti-HA and then SDS-PAGE. Quantifications of three independent western blots (IB) are shown in the bottom. Intensity of gel bands of anti-V5 (first row) versus anti-HA (second row) are shown for each Draf-C. Note that compared with Draf-C^WT^ (lane 3), much less Draf-C^Y510E^ (lane 4) was coimmunoprecipitated with Draf-N.

We next explored the molecular mechanisms for the involvement of Y510 phosphorylation in Draf activation. We first tested whether Y510 modification is important for the enzymatic activity of the Draf kinase by mutating Y510 in Draf-C, consisting of the kinase domain only. Surprisingly, in contrast to the full-length Draf (see Figure 3A and 3C), different Draf-C variants immunoprecipitated from S2 cells exhibited comparable in vitro kinase activities and kinetics, and the levels were similar to the activity of full-length Draf^Y510E^ (Figure 4A). Similar results were found for the Draf-C variants purified from bacteria (Figure S5). These results indicate that Y510 is not directly involved in the enzymatic reaction of the kinase or its substrate recognition.

Y510 and Y538 of Draf are equivalent to Y537 and Y565 of human B-Raf, respectively. Based on the crystal structure of B-Raf [28], Y565 is partially buried into the kinase domain (Figure S6), which may explain why Src64B cannot phosphorylate Y538 in Draf. As a conserved amino acid, Y538 may be important for the structure of Draf. Indeed, we found that the Y538F mutation completely abolished the kinase activity of Draf-C in S2 cells (Figure S7). In contrast, Y537 is exposed on the surface of B-Raf kinase domain (Figure S6), and mutating its equivalent in Draf had no effect on the kinase activity of Draf-C (Figures 4A and S7). These results are consistent with the idea that Y510 of Draf is accessible to modification, plays a regulatory role, and yet may not be critical for maintaining the structure of the kinase domain.

Since the full-length Draf differs from Draf-C by the presence of the inhibitory N-terminal regulatory region, the different kinase activities exhibited by full-length and Draf-C proteins with the same Y510 substitutions (cf. Figures 3A–3C and 4A) suggest that Y510 may normally mediate the inhibitory association between the N-terminal regulatory domain and C-terminal kinase domain of Draf, and that mutating Y510 to a charged residue may disrupt Draf N- C-fragment interaction, resulting in an open configuration and exposed kinase domain.

To test this idea, we investigated the ability of Draf-N to bind to different versions of Draf-C by coimmunoprecipitation. It has been shown that overexpressing C-Raf N-terminal fragment inhibits separately expressed C-Raf activity by physical interaction with its C-terminal kinase domain [17]. Consistent with this report, we found that separately expressed Draf-N was indeed able to bind to Draf-C (Figure 4B; lane 3). As predicted, this interaction is impaired by the Y510E mutation (Figure 4B; lane 4), suggesting that the full-length Draf^Y510E^ has reduced self-inhibition and higher basal activity as observed. Thus, phosphorylation of Y510 by Src64B may play an important regulatory role in Draf activation by relieving the autoinhibition of full-length Draf imposed by its own N-terminal regulatory domains.

### Src64B Is Required for Signaling by Multiple RTKs In Vivo

To test whether endogenous Src64B is generally required for Draf activation in vivo, we analyzed the phenotypes of *Src64B* mutants in multiple biological processes that require RTK-Draf signaling. In *Drosophila*, well-characterized RTKs include Torso, epidermal growth factor receptor (EGFR), and Sevenless [46]. We have shown that *Src64B^Δ17^* mutant flies possess reduced Draf kinase activity (see Figure 1B), which can be attributed to lack of tyrosine phosphorylation of Draf by Src64B (see Figure 1C). However, although Draf kinase activity is reduced in *Src64B^Δ17^* mutants, these flies nonetheless do not exhibit any overt phenotypes that can be attributed to lack of Draf activation. To investigate the importance of Src64B in Draf activation in vivo, we examined the role of Src64B in genetic backgrounds in which Draf signaling is reduced. As with the genetic screen in which *Src64B* was identified [30], such genetic backgrounds may be more sensitive to a reduction in Src64B activity. To this end, we generated double-mutant combinations between *Src64B^Δ17^* and mutant alleles of *torso*, *Draf*, and *Egfr*, and examined the phenotypic consequences in a few RTK-mediated developmental processes in which the requirement for Draf has been well defined. Although we were unable to detect any phenotypic defects in the Torso system (unpublished data), we were able to show that *Src64B* mutation compromised signaling by EGFR and Sevenless (see below). The different outcomes of these genetic tests possibly reflect a different threshold requirement for Draf activation and/or functional redundancy among Src proteins (see Discussion).

**Figure 5:**
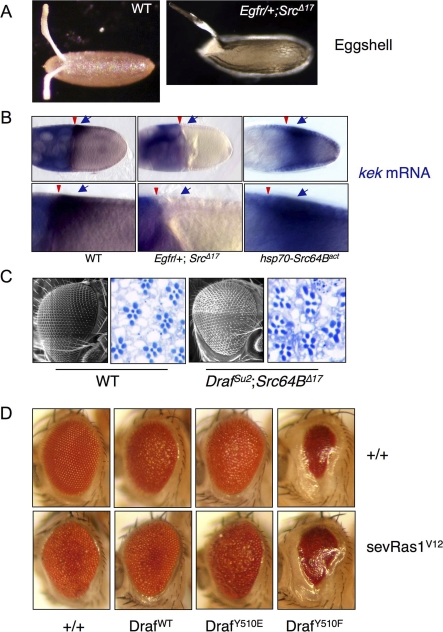
Src64B Is Required for Multiple RTK Pathways during Development (A and B) Src64B is involved in EGFR signaling during oogenesis. (A) Wild-type (WT) eggs have two dorsal appendages arising from the dorsal anterior of the eggshell. The space between these two dorsal appendages represents the dorsal-most cells, which are specified by EGFR signaling. Eggs from *Egfr/+; Src64B^Δ17^* females exhibit a single dorsal appendage (or fusion of two appendages) due to the lack of the dorsal-most cells (“ventralized” phenotype). (B) Expression pattern of the EGFR target gene *kek* in follicle cells of stage 10 egg chambers were detected by in situ hybridization (blue stains and indicated by arrow). EGFR-independent expression of *kek* in nurse cells (diffuse blue stain) is the internal control for staining. The oocyte is located to the right and is surrounded by somatic follicle cells. The nurse cells are located to the left of the egg chamber. Arrowheads point to the boundary between nurse cells and the oocyte. (Left) In the wild-type egg chamber, *kek* is expressed in a gradient in the follicle cells abutting the dorsal-anterior region of the oocyte (arrow). (Middle) The dorsal follicular *kek* expression in *Egfr/+; Src64B^Δ17^* egg chambers is not detectable (arrow). (Right) Expression of Src64B^act^ resulted in increased *kek* expression, such that *kek* is expressed in expanded domains extending to the ventral region. Higher magnifications of the region of *kek* expression in dorsal follicle cells as shown in the bottom. (C) Src64B is involved in R7 cell specification during eye differentiation. (i) Scanning electron micrograph and a section of the compound eye are shown for wild type and *Draf^su2^ Src64B* double homozygotes (right). Photoreceptor cells were stained dark blue. Note that the compound eyes of *Draf^su2^ Src64B* double homozygous mutants are slightly smaller and rough. In the wild-type eye (left), each ommatidium contains seven photoreceptors (R1–R7). R7 is located in the center, which is specified by the Sev RTK pathway. In *Draf^su2^ Src64B* double mutants (right), some ommatidia are missing R7 cells (*n* = 97/438 ommatidia). (D) Effects of expressing Draf variants on eye development. Top row: eyes of *GMR-Gal4, UAS-Draf* transgenes flies. Note that expressing Draf^WT^ and Draf^Y510E^ led to rough eyes, and expressing Draf^Y510F^ caused much reduced eye size. Bottom row: eyes of flies carrying *GMR-Gal4, UAS-Draf* transgenes and *sev-Ras^V12^*. Note that *sev-Ras^V12^* and Draf^WT^ mutually suppressed each other, in agreement with the previous finding that overexpressing Draf^WT^ has dominant-negative effects [61]; Draf^Y510E^ enhanced *sev-Ras^V12^* phenotypes (eyes blistered); Draf^Y510F^ is epistatic to *sev-Ras^V12^*.

During oogenesis, EGFR-Draf signaling is required for specifying the dorsal anterior cell fates in the follicular epithelium of the egg chamber [10]. Reductions in EGFR or Draf gene activities cause ventralization of the egg chamber, resulting in fusion or missing of the pair of dorsal appendages of the eggshell as well as a reduction in the expression of the EGFR target gene *kekkon* (*kek*) (Figure 5A and 5B) [47,48]. *Egfr* heterozygotes (*Egfr*/+) do not exhibit any discernable abnormalities and were indistinguishable from wild type in eggshell morphology (unpublished data) or *kek* expression (Figure 5B). *Src64B^Δ17^* homozygous females lay fewer and smaller eggs [30,36], presumably due to a disruption in the cytoplasmic transfer from the nurse cells to the oocyte during oogenesis. These eggs, however, showed normal spacing between the pair of dorsal appendages (unpublished data; also see [36], indicating that EGFR signaling was normal in *Src64B^Δ17^* egg chambers. In contrast, females heterozygous for *Egfr* and homozygous for *Src64B^Δ17^* (*Egfr/+; Src64B^Δ17^*) laid eggs that were ventralized in 100% of them (*n* > 500; Figure 5A), suggesting a deficiency in EGFR signaling. We next examined the expression of the EGFR target gene *kek*. In wild-type as well as in *Src64B^Δ17^* homozygous or *Egfr* heterozygous flies, *kek* is expressed in a gradient with the highest levels in the dorsal anterior region of the follicle cell layer overlying the oocyte nucleus (Figure 5B, left; unpublished data) [47]. Consistent with the ventralized phenotype of *Egfr/+; Src64B^Δ17^* animals, *kek* expression in the dorsal anterior region of stage 10 egg chamber was undetectable (Figure 5B, middle). Conversely, expression of a *Src64B^act^* transgene, which encodes a constitutively active form of Src64B [31], resulted in expansion of *kek* expression domain to the ventral region of the follicle layer (Figure 5B, right). Thus, Src64B is required for the expression of the EGFR target gene *kek* and patterning the dorsal appendages of the eggshell.

We next investigated whether Src64B also plays a role in mediating signaling from the RTK Sevenless in photoreceptor differentiation during eye development (Figure 5C). It has been shown that Sevenless (Sev) signaling is required for specifying the R7 photoreceptor cell fate [49]. A loss of Sev or a reduction in Ras1 or Draf function results in the loss of R7, and overactivation of these molecules leads to supernumerary R7 phenotype [49]. *Src64B^Δ17^* homozygotes have normal eyes and all the ommatidia in eye sections were of normal morphology and were indistinguishable from those of wild type (*n* > 600 ommatidia; unpublished data). Normal morphology and the presence of R7 were also found in *Draf^Su2^* homozygotes (unpublished data; also see [50]. However, *Draf^Su2^*; *Src64B^Δ17^* double homozygotes exhibit slightly smaller and rougher eyes; their eye sections revealed unequal spacing, and portions of the ommatidia are missing R7 (Figure 5C, right). It has previously been shown that expression of Src64B^act^ can induce ectopic R7 photoreceptor formation [31] and ectopic expression of the Torso target gene *tll* [30], and expression of a dominant-negative Src64B results in loss of R7 cells [31]. Thus, Src64B is required for Sev RTK signaling.

Moreover, we investigated the effects of expression Draf variants on eye development. When expressed by the eye-specific drive *GMR-Gal4*, Draf^WT^ and Draf^Y510E^ caused rough eye phenotypes, whereas Draf^Y510F^ led to a much-reduced eye size (Figure 5D, top row). To understand these eye phenotypes with regard to Draf activity, we expressed the Draf variants in the background of Ras1^V12^ expression. Ras1^V12^ overactivates Draf and causes rough eyes [51]. We found that Draf^WT^ partially suppressed, Draf^Y510E^ enhanced, and Draf^Y510F^ was epistatic to the rough eye phenotype due to Ras1^V12^ expression (Figure 5D, bottom). These results are consistent with the interpretation that Draf^WT^ was slightly and Draf^Y510F^ strongly dominant-negative, whereas Draf^Y510E^ had elevated kinase activity.

We further found that expression of Src64B^act^ in the wing imaginal disc causes extra vein formation (unpublished data) and activation of Rolled/ERK (Figure S8). Taken all together, these results strongly suggest that Src64B functions in multiple developmental processes that require RTK signaling, consistent with a role in Draf activation in vivo.

## Discussion

We have investigated the mechanism of Draf activation by both genetic and biochemical means. We have identified a novel tyrosine residue Y510 within the Draf kinase domain that mediates Draf phosphorylation by Src64B and is important for Draf activation in vivo. Substitution of Y510 to glutamate results in activation of the full-length Draf, without affecting a truncated Draf-C. These results suggest that Y510 phosphorylation plays an important regulatory role in Draf activation by relief of autoinhibition. Since Y510 is conserved among all Raf proteins, this mechanism of Raf activation by Src might be evolutionarily conserved.

### The Mechanism of Raf Activation Mediated by Y510 Phosphorylation

**Figure 6:**
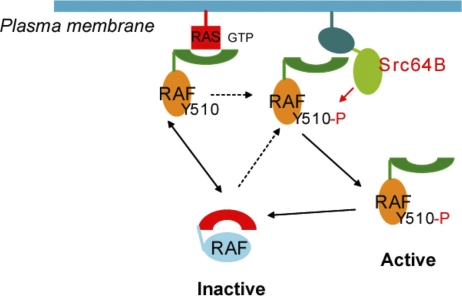
A Proposed Model for Regulation of Draf by Src64B Inactive Raf assumes a folded or “closed” conformation due to association of the N-terminal regulatory domains with the C-terminal kinase domain. Binding to Ras-GTP transiently dissociates the N-terminus from the C-terminus, forming to an “open” conformation and exposing Y510. Subsequent phosphorylation of Y510 by Src prevents the re-association of the N- and C-termini, stabilizing the “open” conformation of Raf, allowing its further modification or association with other proteins.

In light of our results, we propose the following general model to account for Raf activation by phosphorylation (Figure 6). In the inactive state of Raf, Y510 may participate in physical interaction between the N-terminal regulatory domains and the C-terminal kinase domain. Binding to Ras by the N-terminus will transiently dissociate it from the C-terminus, forming to an “open” conformation and exposing Y510. Subsequent phosphorylation of Y510 will prevent a reassociation of the N- and C- termini, stabilizing the “open” conformation of Raf. This model is supported by results of our mutagenesis studies. Mutations in Y510 in the context of Raf C-terminus have minimal effects on Raf kinase activity. In contrast, mutating Y510 to an acidic residue (glutamate) in full-length Raf could result in a static electrical hindrance similar to the effects of phosphorylation, thereby preventing the formation of the inactive, “closed” Raf configuration and leading to Raf activation. Phosphorylation as a means to interfere with interaction between protein domains has been documented for Raf and other proteins [17,21,41–43].

### Src64B Phosphorylates and Activates Draf

It has previously been shown that expressing activated Src in cultured mammalian cells leads to C-Raf activation and a concomitant phosphorylation on Y340 and/or Y341 [15,21–23], and that purified Src can directly phosphorylate Y341 in vitro [52]. Y340 and Y341 immediately follow two serine residues (S338 and S339). Together, these residues constitute the N-region that appears important for C-Raf regulation [6,11]. In Draf, the positions equivalent to Y340/341 of C-Raf are occupied by two glutamate residues. The sequence SSEE in the N-region of Draf is thus more similar to the arrangement in B-Raf, which has SSDD occupying these positions. Since it has been reported that Src does not activate B-Raf [15], the sequence similarity between Draf and B-Raf has raised the issue of whether Draf can be phosphorylated or activated by *Drosophila* Src at all. However, results from our genetic and biochemical analyses of Src64B and Draf activation in *Drosophila* contrast the conclusion concerning B-Raf activation and indicates that Src64B may directly activate Draf by phosphorylating Y510, a conserved tyrosine residue located in the kinase domain of all Raf proteins. Since Y510 is conserved in all Raf proteins, but not in other kinases other than Ksr (whose kinase domain is mostly similar to Raf in sequence), we propose that the equivalent of Y510 in Raf kinase domain may serve as a key residue mediating Raf phosphorylation and activation by Src.

### Biological Functions of Src64B during *Drosophila* Development

Genetic studies of Src64B suggest that different biological processes may require different threshold levels of Src64B function. Mutagenic studies intended to isolate loss-of-function alleles of *Src64B* have so far resulted in a few weak alleles that do not affect viability [36]. However, animals with reduced *Src64B* function, such as *Src64B^Δ17^* homozygotes, are partially female sterile and are associated with defective ovarian ring canal morphogenesis [36]. We detected different degrees of disruption in RTK signaling in *Src64B* mutant flies only in conditions in which the RTK pathway is compromised, such as in combination with viable *Draf* mutants or *Egfr* heterozygotes. Ovarian ring canal morphogenesis probably requires the highest amount of Src64B and becomes the first process to fail when Src64B function is reduced. Interestingly, we also observed different threshold requirement for Src64B in different RTK signaling pathways. Among the best characterized biological processes that require RTK/Draf signaling, the dorsoventral polarity of the egg chamber appears the most sensitive; we found a 100% penetrant ventralization phenotype in *Egfr/+*; *Src64B^Δ17^* flies. *Draf^Su2^; Src64B^Δ17^* double homozygous flies also exhibit a 100% eggshell ventralization, and *Draf^Su2^* homozygotes alone exhibit a certain degree of eggshell ventralization (unpublished data). The SEV pathway appears less sensitive to reductions in Src64B/Draf activity, such that approximately 22% of the *Draf^Su2^; Src64B^Δ17^* double homozygous flies are missing R7. The least-affected RTK pathway is the Torso pathway, which utilizes the same Ras1/Draf signaling cassette to specify the embryonic terminal structures [33]. However, in none of the above mutant combinations did we detect defects in the Torso pathway (unpublished data). Thus, genetic studies based on a partial loss-of-function *Src64B* allele, *Src64B^Δ17^*, suggest a differential threshold requirement for Src64B function in the following biological processes: ring canal formation > egg chamber dorsoventral polarity > R7 specification > embryonic termini formation. Since Draf^Y510F^ poorly rescued the terminal structure in *torso* null embryos, tyrosine phosphorylation on Y510 could be conferred by protein kinases other than Src64B.

### Possible Redundancy of *Drosophila* Src Family Tyrosine Kinases

It is possible that Src64B is functionally redundant with other cytosolic tyrosine kinases such as Src42A and Tec29A. This may explain the subtle phenotypes of Src64B mutant alleles. Src42A is another Src homolog in the fly genome. Outside of the Src family, Tec kinases are mostly homologous to Src kinases. In *Drosophila*, Tec29A has been identified that functions downstream from Src64B during oogenesis [36,53]. Moreover, Tec29A is also a potential Draf activator [30]. It is thus an interesting scenario that these three tyrosine kinases may function redundantly in phosphorylating and activating Draf. However, it has been shown that the kinase activity of Src42A is not crucial for mediating RTK signaling, as overexpression of either a wild-type or kinase-dead form of Src42A equally induces hyperactivation of RTK signaling [54]. It has recently been shown that a kinase-independent scaffolding function of Src42A regulates Draf by a novel CNK-dependent derepression mechanism, and Src64B does not share such a function [55]. Thus, Src64B and Src42A may have overlapping as well as distinct functions, and further investigation is required to determine how these tyrosine kinases are involved in Draf signaling.

## Materials and Methods

### Fly strains and genetics.

The following strong or null alleles were used in this study: *torso^XR1^* [56], *Ras1^ΔC40B^* [35], *Draf^11−29^* [57], *Draf^C110^*, *Draf^Su2^* [50], *Src64B^Δ17^*, and *Src64B^PI^* [36]. *Egfr^f2^*, *sev-Ras^V12^* (on a TM3 balancer chromosome), *Nanos-Gal4* (on X), *GMR-Gal4* (on the second chromosome), and *hsp70-Gal4* are from the Bloomington *Drosophila* Stock Center. Transgenic lines carrying *hsp70-Src64B^act^*–expressing heat-shock–inducible, activated Src64B and Draf were previously described [31]. The dominant female sterile (DFS) technique [58] was used to generate germline clone (GLC) embryos that lack the maternal product of *Ras1* and *Draf*. Embryos lacking the maternal *torso* product were produced by *torso^XR1^* homozygous females. Standard techniques were used to produce transgenic flies carrying different *UAS-Draf* variants in the pUAST vector [45].

### Plasmids and antibodies.

His-tagged Draf and Draf-C (a.a. 368–739) constructs were made by ligating Draf cDNA fragments to pQE vectors (Qiagen). Mutations of Y510 and Y538 were introduced by PCR and verified by sequencing. Draf-N (a.a. 1–367) and Draf-C were subcloned into the HA-pUAST and Flag-pUAST vectors. GST-tagged Src64B^act^ was made by ligating a Src64B cDNA fragment encoding a.a. 1–540 to the pGEX-KG vector. Non-tagged Src64B^act^ and full-length Draf variants were cloned into the pUAST vector. GST-tagged Dsor and the kinase-dead Draf (K497M) were generous gifts from E. Hafen and L. Ambrosio, respectively. V5-Draf and V5-Src64B^act^ were made by ligating PCR-amplified cDNA fragments to the pMT-V5 vector (Invitrogen).

Antibodies specific for phospho-MEK1/2, MEK1/2, and phosphotyrosine (pY102) were from Cell Signaling Technology and were used at 1:1,000 dilution. Anti-Draf serum (gift from D. Morrison), anti-Src64B (gift from J. Cooper), mouse anti-V5 (Invitrogen), mouse anti-Flag (Sigma), and rat anti-HA (Roche) were used at 1:1,000. Rabbit anti-V5 (QED) and goat anti-HA (QED) were used to immunoprecipitate tagged proteins. Rabbit polyclonal antibody against phospho-Y510 of Draf (Y510) was produced by immunizing rabbit with the phospho-peptide CEGSSLpYKHVHVS, which represents the amino acid sequence around Draf Y510 (underlined). Antibody production and affinity purification were carried out by Proteintech Group.

### Transgene expression and histochemistry.

To determine the effects of heat-shock expression of Src64B^act^ on embryogenesis and Torso signaling, 0–1-h-old embryos carrying one copy of *hsp70-Src64B^act^* were collected and were allowed to continue development for an additional hour at 25 °C. They were then subjected to a brief heat shock at 34 °C for 5 min in water bath. Heat-shocked embryos were allowed to develop for 20 min. at 25 °C and then fixed, or allowed to develop for 24 h for cuticle examination. To examine the effects of Src64B^act^ during oogenesis, females containing one copy of *hsp70-Src64B^act^* were heat shocked at 34 °C for 10 min in water bath, allowed to recover for 10 min at room temperature, and then dissected to fix the ovary. Detection of *tll* and *kek* mRNA expression by in situ hybridization was carried out as previous reported [8,47].

### Cell culture and transfection.

*Drosophila* Schneider L2 (S2) cells were cultured at 25 °C in *Drosophila* Serum-Free Media (SFM; Invitrogen) supplemented with 10% Fetal Bovine Serum (FBS; Invitrogen) and 0.5× Antibiotic-Antimycotic (Invitrogen). Cells were cultured at 2.5 × 10^6^/ml prior to transfection. Transfection was performed with Cellfectin (Invitrogen) according to the manufacturer's instructions. An actin-Gal4 plasmid was used to induce expression of pUAST transgenes. To induce expression of genes cloned in pMT-V5 vector, Cu_2_SO_4_ (Sigma) was added to the medium at the final concentration of 0.5 mM 16 h after transfection, and cells were harvested 24 h after induction. S2 cells were harvested in the cell lysis buffer (from Cell Signaling Technology).

### In vitro Draf kinase assay in cell-free extracts.

A total of 100 μl of embryos (0–4 h after egg laying) of different genotypes were homogenized in 100 μl of 2× kinase buffer (from Cell Signaling Technology). The lysate was cleared by centrifugation for 10 min at 4 °C. To immunoprecipitate Draf, 2 μl of anti-Draf antibody and 10 μl of Protein A-Sepharose (Sigma) were incubated with 100 μl of embryo lysates overnight at 4 °C. To measure Draf kinase activity, 50 μl of cell-free embryo extracts or immunoprecipitates were mixed with 2 μg of GST-Dsor and 200 μM ATP for 40 min at 30 °C. To deplete Draf from the lysate, 1 μl of anti-Draf antibody and 10 μl of Protein A-Sepharose (Sigma) were added to 100 μl of lysate and the mixture was incubated for 2 h at 4 °C in the presence of 1× protease inhibitor cocktail (Sigma) prior to centrifugation. To assess the effects of Src64B, Draf immunoprecipitates were incubated with 2 μg of GST-Src^act^ for 60 min at 30 °C in the presence of 200 μM ATP in 50 μl of kinase buffer. The reaction mixture was centrifuged, and the Draf immunoprecipitates were washed three times and were then subjected to in vitro Draf kinase assay. Draf kinase activity was detected as Dsor phosphorylation by anti-pMEK following SDS-PAGE.

### Protein production and in vitro kinase assays using purified proteins.

His-Draf and His-Draf-C variants, GST-Src64B^act^, and GST-Dsor were purified from Escherichia coli BL-21 by standard affinity purification. To purify His-Draf variants (the bulk of which was insoluble), E. coli cells were grown at 28 °C, protein expression was induced by IPTG when cell density reached an optical density (O.D.) of 0.8, and cells were harvested 30 min following induction. Four liters of clear cell lysate (containing a low concentration of soluble His-Draf) was incubated with 2 ml of Ni-NTA agarose overnight at 4 °C in the presence of ß-mercaptoethanol. Soluble His-Draf protein was eluted from Ni-NTA agarose pellet with 8 ml of elution buffer, dialyzed, and further concentrated by using BIOMAX centrifugal filter (Millipore) to a 30-μl final volume. Substrate proteins were dephosphorylated by alkaline phosphatase (Promega) prior to kinase assays. Kinase assays for bacterial proteins were carried out by mixing 1 μg of each of the kinase and substrate into 50 μl of kinase buffer in the presence of 1 mM ATP at 30 °C for 6 h (for Dsor as substrate) or 15 h (for Draf as substrate).

### Expressing Draf proteins in early embryos by mRNA microinjection

mRNA microinjection was performed as previously described [8]. Draf mRNA was synthesized using the mMESSAGE mMACHINE T7 Kit (Ambion) and was injected from the posterior at 1 μg/μl into syncytial blastoderm-stage embryos of desired genotypes. The effects of expressed Draf protein on Torso signaling were analyzed with injected *torso* and *Draf* germline clone embryos.

### Sequence analysis and comparison.

Protein sequence alignment of Raf family members were generated by the ClustalW program (http://www.ebi.ac.uk/clustalw/index.html).

## Supporting Information

Figure S1 Raf Family Proteins and Regulatory Residues Schematic representations of C-Raf, B-Raf, and *Drosophila* Draf. The S338 and Y341 are phosphorylated upon C-Raf activation by Ras-GTP. S417 in Draf and S445 in B-Raf are constitutively phosphorylated. Y341 in C-Raf is replaced by acid amino acids in Draf and B-Raf [6,29].
(137 KB JPG)

Figure S2 Activity of GST-Src64B^act^ Purified from Bacteria GST-Src64B^act^ was expressed in E. coli by standard IPTG induction. Crude lysate was subjected to SDS-PAGE and stained with Coomassie blue (left) or transferred to membrane and blotted with anti-pTyr (middle), or further purified by glutathione beads (unpublished data). A total of 1 μg of purified GST-Src64B^act^ was mixed with *Drosophila* embryo extracts for 30 min, and the mixture was subjected to SDS-PAGE and blotted with anti-pTyr (right). Note the increased tyrosine phosphorylation in E. coli and embryo extracts in the presence of GST-Src64B^act^.
(503 KB JPG)

Figure S3 Expression of Src64B^act^ Promotes Viability of *Draf^C110^* Mutants Draf^C110^ contains an R217L mutation that disrupts its binding to Ras, and *Draf^C110^* hemizygous males die at the late pupae stage. [7,8,50,59]. When animals were raised at 28 °C, one copy of the *hsp70-Src64B^act^* transgene rescued 6.45% of *Draf^C110^* hemizygous males to viable adult flies. For comparison, one copy of *hsp70-Draf-C^WT^* transgene [8,9] rescued 34.9% of *Draf^C110^* hemizygous males to viability. Percent viability is the number of surviving *Draf^C110^* hemizygous males versus the total number of progeny flies scored. The statistical significance of the differences was analyzed by chi-square tests. *p* Values are indicated.
(277 KB JPG)

Figure S4 Titers of Anti-pY510 Sera The Draf phospho-peptide CEGSSLpYKHVHVS surrounding Y510 was used to inject rabbits for antibody production. Antibody production and affinity purification were carried out by Proteintech Group. ELISA results were supplied by Proteintech Group.
(1.92 MB JPG)

Figure S5 Y510 Is Not Essential for Draf Enzymatic Activity His-tagged Draf-C^WT^, Draf-C^Y510F^, and Draf-C^Y510E^ were expressed in E. coli and then purified by Ni beads. Purified proteins (1 μg) were used to perform in vitro kinase assays using bacterially expressed GST-Dsor as substrate. The kinase activity was measured by anti-pMEK in western blots.
(166 KB JPG)

Figure S6 The Draf Y510 and Y538 Equivalents in B-Raf (A) The hydroxyl group of Y537 (equivalent to Y510 in Draf) is exposed on the surface of B-Raf kinase domain. The Protein Data Bank (PDB) code of the B-Raf structure is 1UWH [28].
(B) The hydroxyl group of Y565 (equivalent to Y538 in Draf) is buried into the B-Raf kinase domain.
(C) Positions of Y537 and Y565 relative to the P-loop (in green) and activation segment (in yellow), two regions known to be important for B-Raf activation, are shown.
(D) Y537 (in red), P-loop, and the activation segment are adjacent on the 3-D structure of the B-Raf kinase domain.
(914 KB JPG)

Figure S7 Y538 Is Required for Draf-C Kinase Activity Expression of Draf-C^WT^ or Draf-C^Y510F^, but not Draf-C^Y538F^, increased the phosphorylation levels of Dsor in S2 cells. Since Y538 is highly conserved in all protein kinases (see Figure 2C), it might be important for the structure and/or enzymatic activity of the kinase domain.
(164 KB JPG)

Figure S8 Expressing Src64B Increases ERK Activation ERK activation in third instar imaginal discs was detected by anti-dpERK. (A) wild-type pattern of dpERK indicating the positions of future vein cells. (B) in a *rho vn* double mutant background, dpERK expression is much reduced, especially in the ventral region (arrow in [A and B]; also see [60]). Heat-shock induction of Src64B^Δ540^ caused widespread ERK activation, such that the whole wing pouch was positively stained by the anti-dpERK antibody (C and D). Significantly, the ectopic ERK activation induced by Src64B^Δ540^ was not affected by the absence of *rho* and *vn*, as in *rho vn* double-mutant wing discs, similar expansion of dpERK domains was found.
(692 KB JPG)

### Accession Numbers

The FlyBase (http://flybase.bio.indiana.edu) accession numbers for the genes mentioned in this paper are as follows: *Draf* (FBgn0003079), *Ras* (FBgn0003205), *Src64B* (FBgn0003501), and *Torso* (FBgn0003733).

The GenBank (http://www.ncbi.nlm.nih.gov/Genbank) accession numbers are as follows: Anopheles gambiae raf homolog (EAA13186), A-Raf (TVHUAF), B-Raf (TVHUBF), Btk29A (AAF52631), CG8789 (AAF49129)C-Raf (TVHUF6), CTR1 (AAA32779), Draf (AAF45774), Egfr (AAM70919), ksr (AAF52021), LIMK1 (AAF48176), lin-45 (AAA28142), PhKgamma (AAG22343), and Src64B (AAF47922).

The Protein Data Bank (http://www.rcsb.org/pdb) code of the B-Raf structure is 1UWH.

## Abbreviations

a.a.: amino acid
Draf: *Drosophila* Raf homolog
Dsor: downstream suppressor of Raf
EGFR: epidermal growth factor receptor
ERK: extracellular signal-regulated kinase
MEK: mitogen-activated protein kinase kinase
RTK: receptor tyrosine kinase
SFK: Src family kinase
